# Aberrantly hypermethylated ARID1B is a novel biomarker and potential therapeutic target of colon adenocarcinoma

**DOI:** 10.3389/fgene.2022.914354

**Published:** 2022-10-14

**Authors:** Salem Baldi, Yun He, Igor Ivanov, Hassan Khamgan, Mohammed Safi, Mohammed Alradhi, Abdullah Shopit, Abdullah Al-Danakh, Murad Al-Nusaif, Yaping Gao, Hui Tian

**Affiliations:** ^1^ Research Center of Molecular Diagnostics and Sequencing, Axbio Biotechnology (Shenzhen) Co.,Ltd, Shenzhen, Guangdong, China; ^2^ Department of Molecular Diagnostics and Therapeutics, Genetic Engineering and Biotechnology Research Institute, University of Sadat City, Sadat, Egypt; ^3^ Department of respiratory, Shandong Second Provincial General Hospital, Shandong University, Jinan, China; ^4^ Department of Urology, The Affiliated Hospital of Qingdao Binhai University, Qingdao, China; ^5^ Academic Integrated Medicine and Collage of Pharmacy, School of Pharmacy, Department of Pharmacology, Dalian Medical University, Dalian, China; ^6^ Department of Urology, First Affiliated Hospital of Dalian Medical University, Dalian, China; ^7^ Center for Clinical Research on Neurological Diseases, First Affiliated Hospital, Dalian Medical University, Dalian, China; ^8^ Research Center of Molecular Diagnostics and Sequencing, Research Institute of Tsinghua University in Shenzhen, Shenzhen, Guangdong, China

**Keywords:** ARID1B, DNA Methylation, RNA expression, Immune Cell Infiltration, Colon adenocarcinoma, TCGA

## Abstract

**Background and Objective:** Understanding the tumor microenvironment (TME) and immune cell infiltration (ICI) may help guide immunotherapy efforts for colon cancer (COAD). However, whether ARID1B is truly regulated by hypermethylation or linked to immune infiltration remains unknown. The current work focused on the ARID1B gene expression and methylation in COAD, as well as its relation with ICI.

**Methods and Results:** Multiple tools based on TCGA were used to analyze the differences in the expression of the ARID1B gene, DNA methylation, and its association with various clinicopathological features, somatic mutations, copy number variation, and the prognosis of patients with COAD. According to the analysis results, patients with high mRNA, low methylation levels showed better overall survival than patients with low mRNA, high methylation levels. The correlation analysis of immune cell infiltration and immune checkpoint gene expression showed that the infiltration rates of the main ICI subtypes, cancer-associated fibroblast, and myeloid cells were significantly enriched and correlated with ARID1B in COAD. An association between ARID1B expression and immune infiltration in COAD was found by correlating ICI indicators with ARID1B expression in the immune cell composition of the COAD microenvironment. Notably, M2 chemokines were related to ARID1B expression, while M1 chemokines were not.

**Conclusion:** This study provided evidence that ARID1B may have a role in the pathogenesis of COAD. The specific underlying mechanisms that could be responsible for ARID1B’s downregulation in COAD will need to be investigated in the future.

## Introduction

Colon cancer is a prevalent and worldwide malignant tumour due to the late diagnosis (Wang et al., 2020; Siegel et al., 2022). Colon adenocarcinoma (COAD) is also one of the most cancers that are affected by intertumoral heterogeneity due to immunosuppression factors, and, immune cell subset dysfunction such as M2 macrophage polarization, CD8^+^ T cells, B cells, and natural killer cells (Zhang et al., 2020a). The main epigenetic modifications of gene expression include DNA methylation, histone modification, chromatin remodelling, and RNA regulation that can influence gene transcription processes related to cell activity, which ultimately produce tumours (Han and Yoon, 2012; Cheng et al., 2021; Zebley et al., 2021). DNA methylation leads to changes in chromatin structure, DNA conformation, DNA stability, and the way that DNA interacts with proteins, thereby controlling gene expression and cause cancer (Li et al., 2014) As mentioned in the literature review, colon cancer is diagnosed in the middle or late stage and an effective early diagnosis of COAD is limited, therefore, it represents a major cause of death worldwide (Li et al., 2014; Zhou et al., 2019). The important analysis of DNA aberrant methylation comes from its frequent and early occurrence during the initial stage of the tumour that can be used as a potential biomarker for cancer detection, monitoring, and timed treatment (Watts et al., 2008; Salta et al., 2018). The tumor microenvironment (TME) is mainly composed of tumour cells, tumour-infiltrating immune cells, and matrix components (Trikha et al., 2016; Jiang et al., 2020). Based on recent studies, tumour immune cell infiltration (ICI) is related to the sensitivity of immunotherapy and the prognosis of cancers including colon cancer (Oda et al., 2018; Zhang et al., 2020b; Sato et al., 2020). Defects in the chromatin remodelling factor, ARID1B, cause extensive dysregulation across different cancer types (Kadoch et al., 2013; Aso et al., 2015; Tessier-Cloutier et al., 2020). However, the prognosis significance of ARID1B and its methylation in colon cancer need to be clarified. Multiple tools based on TCGA were used, and correlation analysis was performed to assess ARID1B expression, CpG methylation, and its association with various clinicopathological features, somatic mutation, copy number variation, and the prognosis of patients with COAD. The correlation analysis was also carried out between ARID1B and the tumour immune infiltration level in COAD.

## Materials and methods

### ARID1B gene expression and its DNA methylation analysis

The expression level of ARID1B mRNA and protein in normal and tumor COAD tissues was first estimated using the TNMplot online database (https://www.tnmplot.com/) and The Human Protein Atlas (https://www.proteinatlas.org/), respectively (Á and Győrffy, 2021; Uhlen et al., 2017). UALCAN provides promoter DNA methylation data from the TCGA for most of the genes http://ualcan.path.uab.edu/. UALCAN was also used to determine the association between ARID1B methylation and clinicopathological variables in COAD patients, including age, gender, tumor stage, and lymph metastasis (Chandrashekar et al., 2017). MethSurv (https://biit.cs.ut.ee/methsurv/), the third online way, is a web tool to perform multivariable survival analysis using DNA methylation data (Modhukur et al., 2018). Subsequently, the significantly identified probes were tested by univariate and multivariate analysis-based on clinical variables. Additionally, we also explored the expression of DNA methyl transferases (DNMT1and DNMT3A) between ARID1B high and ARID1B low based on TCGA database.

### Cell culture and qPCR analysis

Human colonic epithelial cells (HCoEpiC) and colon adenocarcinoma cell lines (HCT116 and LoVo) were cultured, supplied with 5% CO_2_, and incubated at 37°C. Extraction of total RNA, synthesis of cDNA, and qPCR conditions using SYPR green analysis reagent were previously published (Baldi et al., 2021). ARID1B forward (CAA​TGC​CAC​AGG​AAA​GAG​GTT​T) and reverse (CTG​TCT​GTT​GAG​GTC​CAT​ACT​GA) primers were utilized.

### Survival analysis and clinical value of ARID1B methylation

CanEvolve is a public online tool used to analyze and visualize TCGA clinical and phenotype data. Three gene expression TCGA COAD datasets, including overall survival (OS), disease-free survival (DFS), and disease-specific survival (DSS) were selected from the CanEvolve online tool (http://www.canevolve.org/) (Samur et al., 2013). According to the median cut-off, patients in each dataset were divided into high and low expression groups to reveal the correlation between ARID1B expression and overall survival (OS). Whereas the clinical role of ARID1B methylation in COAD and the relationship between ARID1B methylation and the above-mentioned clinical elements were estimated using UALCAN. Besides, MethSurv [MethSurv-A web tool to perform multivariable survival analysis using DNA methylation data (ut.ee)] also contributed to the connection between position distribution around CpG islands and the prognosis of COAD patients.

### Epigenetic analysis of gene promotor regions of ARID1B

SMART tools were used to explore the relationship between ARID1B methylated sites, somatic mutation and copy number variation (Li et al., 2019).

### TIMER analysis

The reliability and validity of TIMER2 website in the study of immune cell infiltration are supported by previous researches enabled us to estimate the correlation between ARID1B expression and immune subset infiltration levels in the COAD microenvironment (http://timer.cistrome.org/) (Li et al., 2017). TIMER2 was also conducted to determine the power of correlation between ARID1B and immune cell markers, chemokines, and immunomodulator levels in the TCGA COAD microenvironment.

### ARID1B related pathway analysis

GSCAlite using TCGA data was conducted to identify ARID1B pathways in COAD tumor http://bioinfo.life.hust.edu.cn/web/GSCALite/(Liu et al., 2018). On the other hand, UALCAN identified potentially associated and co-expressed genes of ARID1B in the TCGA COAD. The heat map of the top 50 upregulated and downregulated genes was created. To identify ARID1B-associated functions in COAD, we performed Gene Set Enrichment Analysis (GSEA) for each set using OmicBeans tool, (http://www.omicsbean.cn/).

## Results

### TCGA analysis of the clinical prognostic value of ARID1B

**FIGURE 1:**
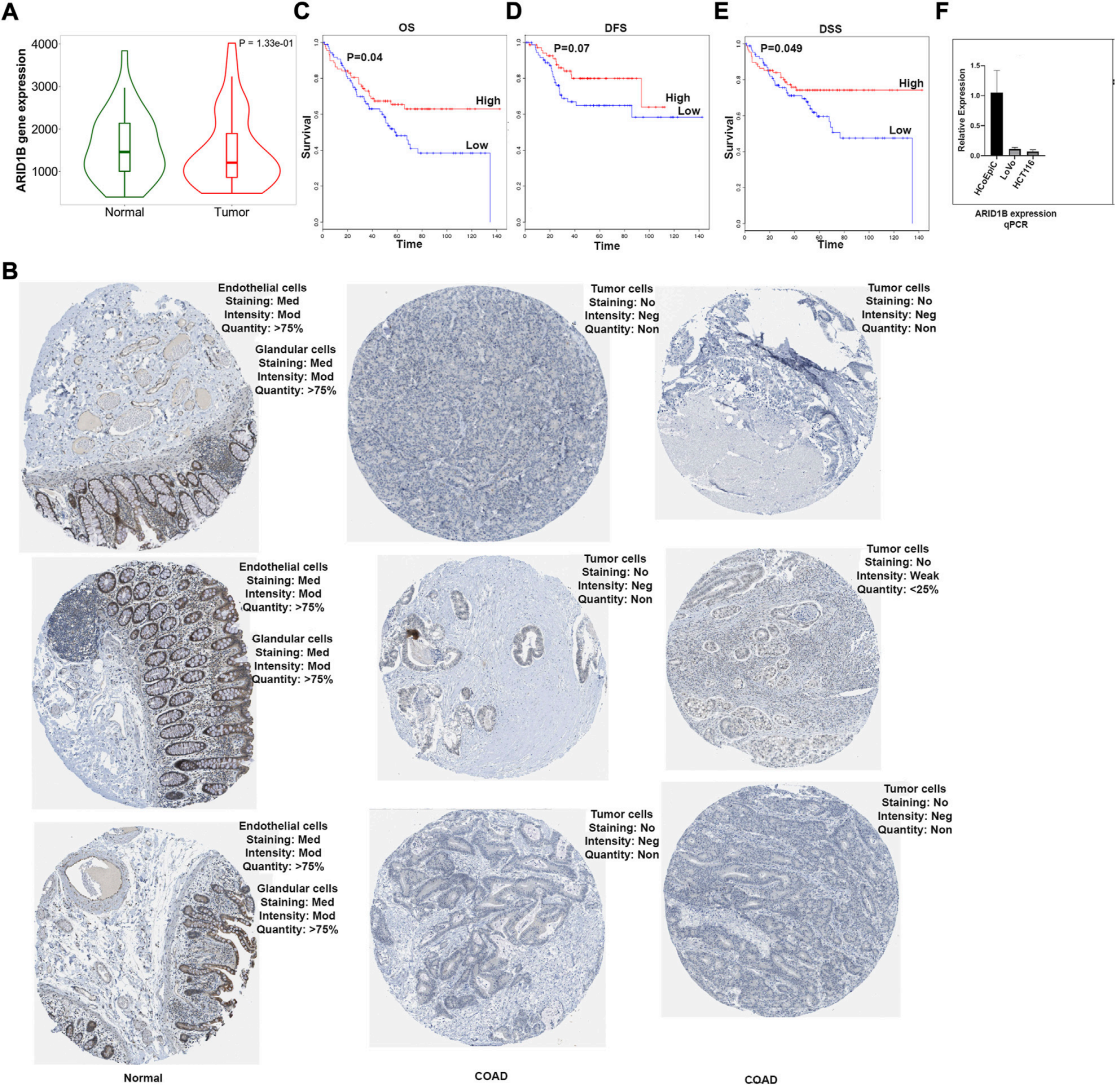
ARID1B mRNA analysis results. **(A)** ARID1B expression from TCGA, **(B)** ARID1B protein expression from HPA, **(C–E)** Kaplan Meir blot of Overall Survival, Disease Free Survival, and Disease Specific Survival. **(F)** qPCR results, ARID1B is downregulated in cancer cell line compared to normal cell line. HPA, Human Protein Atlas; OS, overall survival; DFS, disease-free survival, DSS; disease-specific survival; Med, Medium; Mod, Moderate; Neg, Negative; Non, None.

The first set of questions aimed to measure ARID1B expression levels from the TCGA database. Differential analysis of total of 82 RNA-Seq data using TNMplot database showed that ARID1B had a lower mRNA level in tumor COAD tissues (41 patient) compared to normal tissues (41 patient) (Figure 1A). Furthermore, the HPA tool was used for protein expression analysis and ARID1B protein was decreased in colon tumor cells than endothelial cells and glandular cells (Figure 1B).

### ARID1B is associated with poor prognosis in colon adenocarcinoma

The next question we asked whether ARID1B low expression has a clinical value for COAD patients. The significance of ARID1B gene expression in determining the overall survival (OS), disease-free survival (DFS), and disease-specific survival (DSS) of COAD patients were assessed, and a low level of ARID1B expression was associated with poor prognosis in all three survival analysis datasets (Figures 1C–E). Colon cancer cell lines showed a low mRNA ARID1B expression compared to colon normal cell line (Figure 1F).

### ARID1B is hypermethylated in colon cancer

**FIGURE 2:**
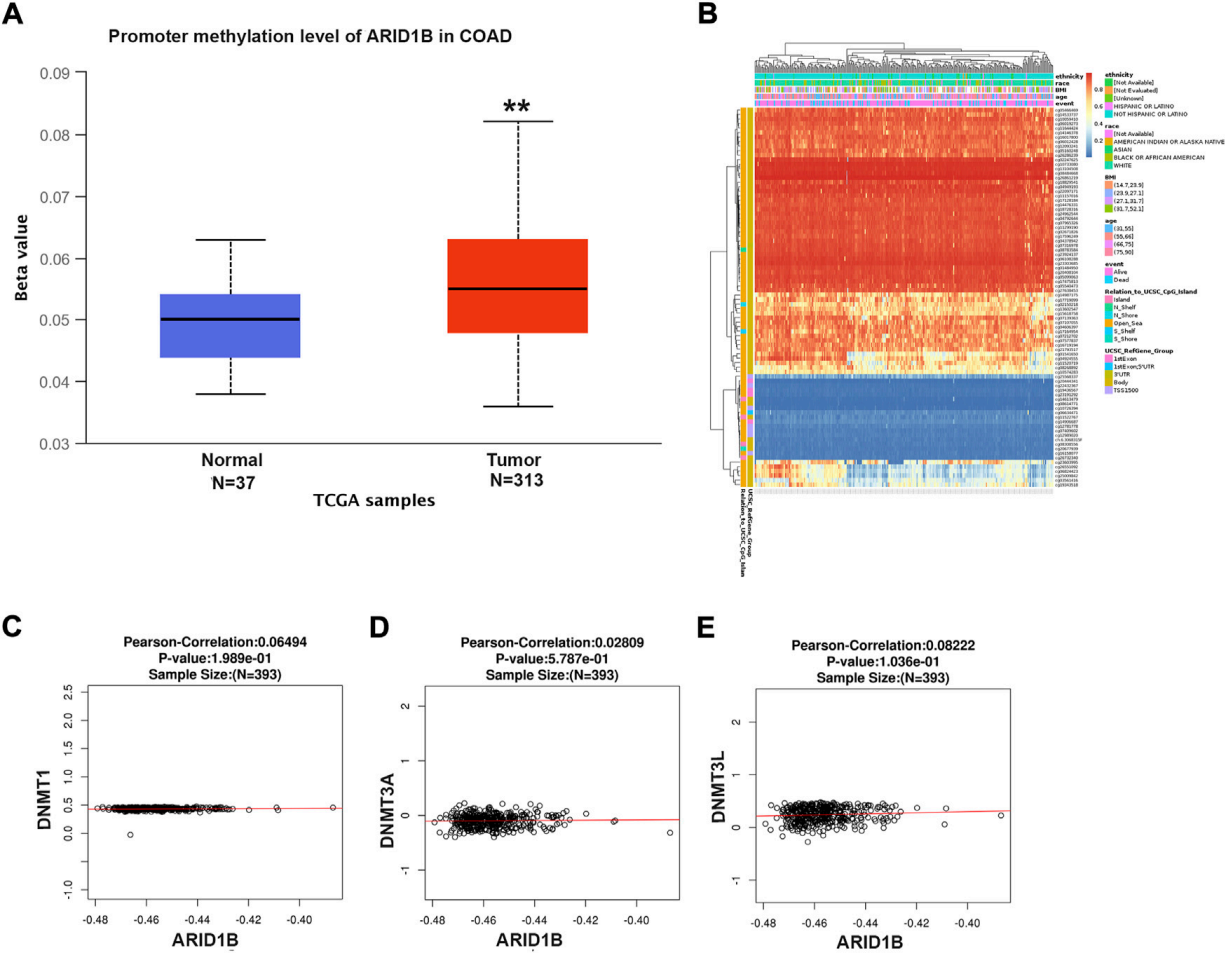
ARID1B methylation level analysis. **(A)** ARID1B methylation level from UALCAN **(B)** Different CpG probs of ARID1B from Methsurv **(C–E)** The positive correlation of ARID1B and methyltransferases. N, Normal; T, Tumor; S, Stage; Cau, Caucasian; Afr-Amr, African, American; Asi, Asian; M, Male, F, Female; y, year; N-W, Normal Wight; E-W, Extreme Wight; Ob, Obese; E-B, Extreme Obese; Ad, Adenocarcinoma; Muc, Mucinous Adenocarcinoma.

To explore the reasons for the low expression of ARID1B in colon cancer, the methylation level of the ARID1B gene was analyzed with UALCAN web tool. The expression of the ARID1B gene in colon cancer was hypermethylated in cancer samples (313) than normal samples (Mu et al., 2018) (Figure 2A). Differentially methylated regions were identified and a heat map was created (Figure 2B). Interestingly, hypermethylation of ARID1B was correlated with the downregulation of ARID1B expression. Additionally, the expression of ARID1B was positively correlated with the expression of three methyl transferases (DNMT1, DNMT3A, and DNMT3L) in colon cancer, although the difference was not significant (Figures 2C–E).

### ARID1B methylation is related to the clinicopathological variables and prognosis of COAD

**FIGURE 3:**
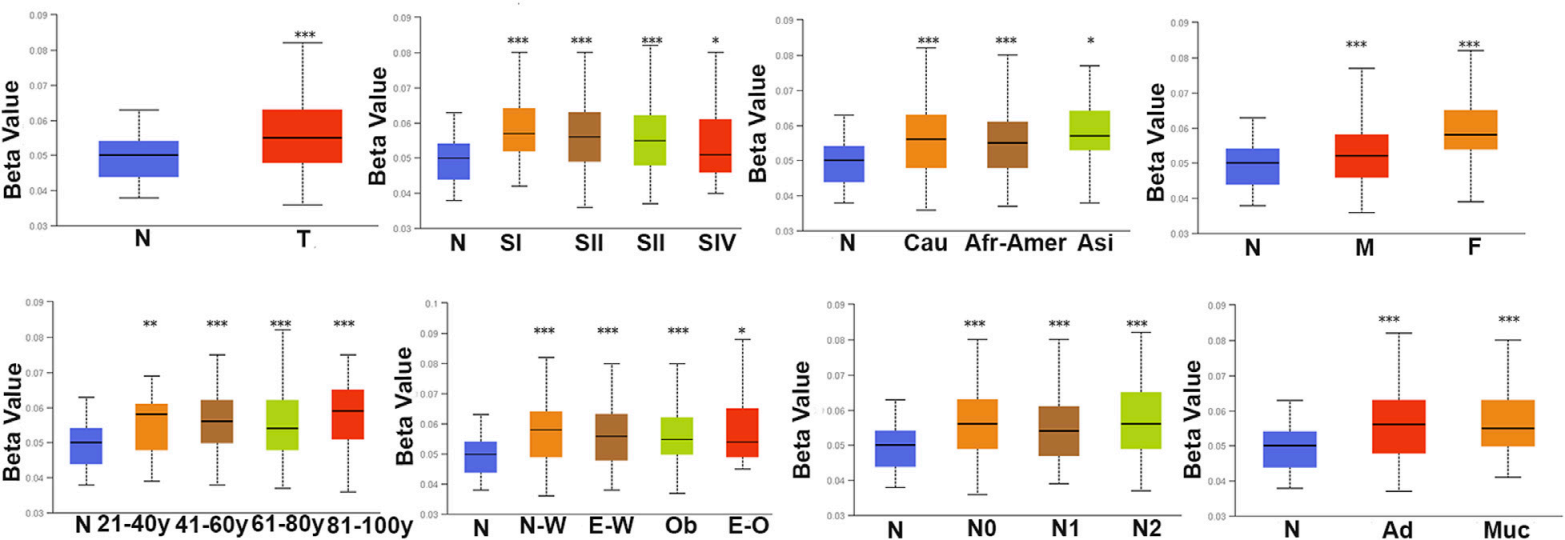
The methylation level of ARID1B across different clinicopathological parameters in COD patients. There is a significant difference between the defined subgroups containing gender, age, race, histological subtype, tumor grade, and nodal metastasis status of patients.

**FIGURE 4:**
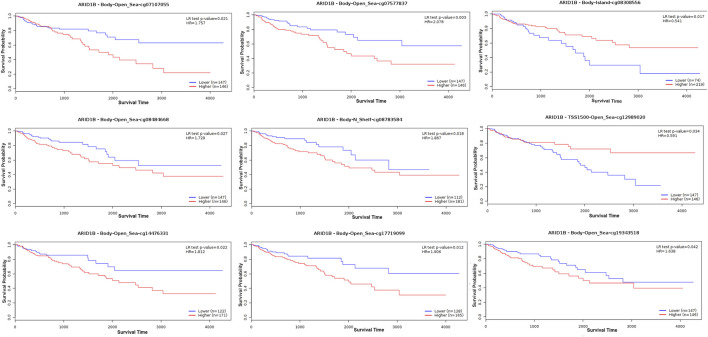
ARID1B hypermethylation impacts the prognosis of COAD patients.

We analyzed the differences in DNA methylation levels in the TCGA COAD cohort and determined the association between ARID1B methylation and clinicopathological variables, including age, gender, tumor stage, lymph metastasis, distant metastasis, and clinical stage. Compared with the normal group, ARID1B was hypermethylated and was associated with all indicated variables as well as clinical with each stage (Figure 3). Significantly, the hypermethylation of ARID1B predicted a shorter overall survival, thus hypomethylation of the ARID1B gene is conducive to survival (Figure 4). This is an interesting consistent result. Univariate and multivariate Cox regression was performed and the data presented in Tables 1, 2 showed that the prognostic significance of hypermethylation was an independent factor of clinicopathological characteristics including age, gender, race, and stage.

**TABLE 1:** Univariate analysis.

Overall survival					
Probe	Hazards. Ratio	CI.Lower	CI.Upper	*p*.value	Z
cg07107055	2.423	0.2873	20.432	0.4159	0.8136
cg07577837	18.6585	0.96	362.6319	0.0532	1.933
cg08484668	17109.36	4.00E-04	7.13E+11	0.2762	1.0888
cg08783584	11971363	4.4493	3.22E+13	0.031	2.1576
cg14476331	253056	0.9222	6.94E+10	0.0515	1.9473
cg17719099	3.4216	0.6514	17.9708	0.1461	1.4536
cg19343518	2.7602	0.7027	10.8419	0.1458	1.4545
cg21793517	181.0568	8.2055	3995.068	0.001	3.2933
cg23603995	1.2343	0.4007	3.8022	0.7138	0.3667

**TABLE 2:** Multivariate analysis.

Variable	Coef	H	Z	95%CI lower	95%CI upper	*p*.value
Age	0.027525	1.027907	2.228524	1.003323	1.053095	0.025846
GenderMALE	0.178965	1.195979	0.586209	0.657443	2.175651	0.557735
RaceBLACK OR AFRICAN AMERICAN	0.252757	1.28757	0.281233	0.221186	7.495211	0.778531
RaceWHITE	−0.15422	0.857081	−0.18606	0.16884	4.350798	0.852397
StageStage II	0.75889	2.135903	1.185325	0.60899	7.491226	0.235889
StageStage III	0.971959	2.643117	1.517938	0.753493	9.271579	0.12903
StageStage IV	2.550279	12.81068	3.915276	3.573783	45.92153	9.03E-05
cg07107055	−2.70048	0.067173	−1.92926	0.004322	1.043918	0.053699
cg07577837	0.921898	2.514057	0.448105	0.044584	141.766	0.654077
cg08484668	0.227981	1.256061	0.023353	6.16E-09	2.56E+08	0.981368
cg08783584	6.649516	772.4103	0.694165	5.42E-06	1.1E+11	0.487579
cg14476331	9.892837	19788.12	1.269733	0.004618	8.48E+10	0.20418
cg17719099	0.271772	1.312288	0.244605	0.148692	11.58163	0.806762
cg19343518	1.370621	3.937793	1.528212	0.678936	22.83901	0.12646
cg21793517	3.309323	27.36659	1.517419	0.380929	1966.063	0.129161
cg23603995	0.059191	1.060978	0.087332	0.281051	4.00523	0.930408

### Functional enrichment and PPI analysis

**FIGURE 5:**
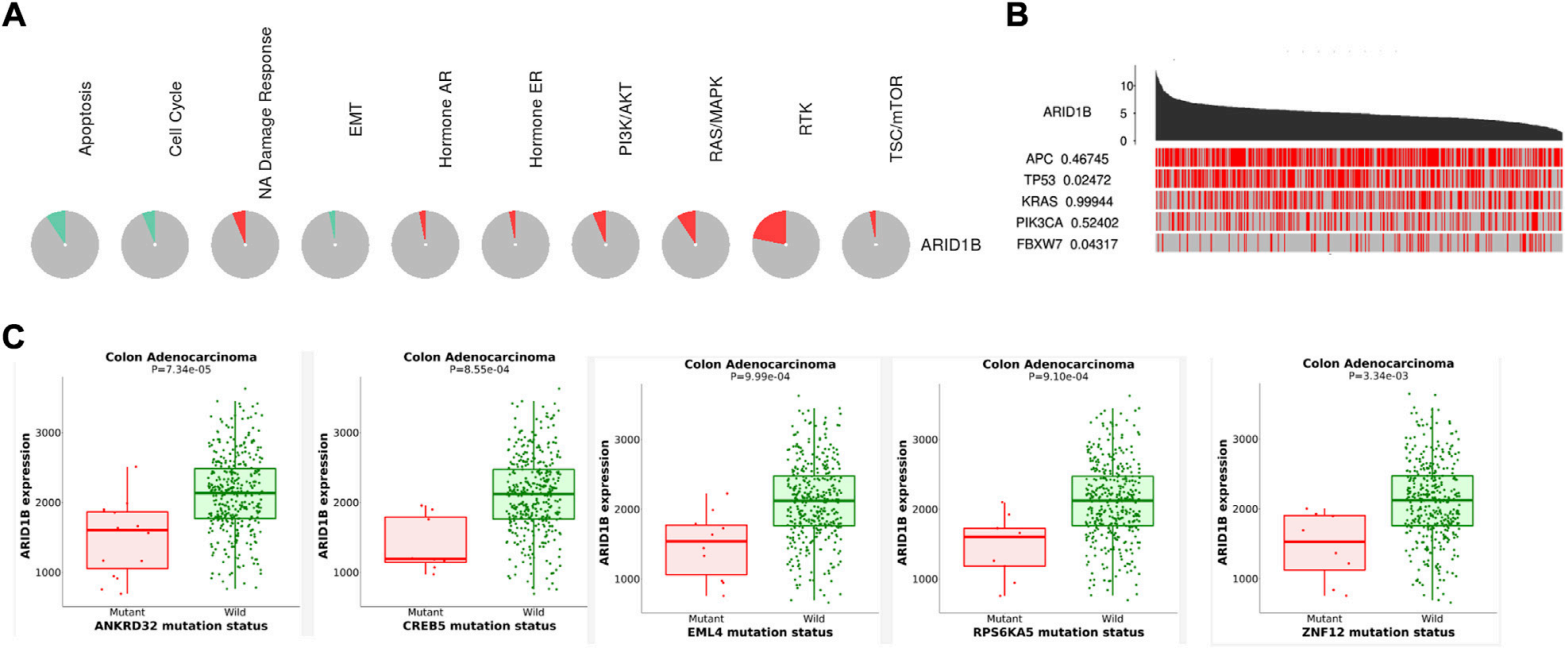
Enrichment pathways of ARID1B. **(A)** ARID1B is inhibited and activated in pathways indicated with green and red colors, respectively **(B)** A permutation test *p*-value of ARID1B between driver mutated and non-mutated samples **(C)** Analysis correlation between ARID1B expression and some mutated genes that cause a low level of ARID1B was explored.

Based on the functional enrichment and correlation analysis with the representative molecules of the pathway, the expression of the ARID1B gene was related to the PI3k/AKT, RTK, NA damage response, Hormone AR, RAS/MAPK, and TSC/mTOR pathways while it was inhibited in apoptosis, cell cycle, and EMT pathways (Figure 5A). Furthermore, the ARID1B gene exhibits high expression when the driving genes (APC and FBXW7) are mutated (Figure 5B). Contrary, there was a list of mutated gene decreased ARID1B expression in COAD (Figure 5C).

### ARID1B methylated sites are associated with somatic variations

**FIGURE 6:**
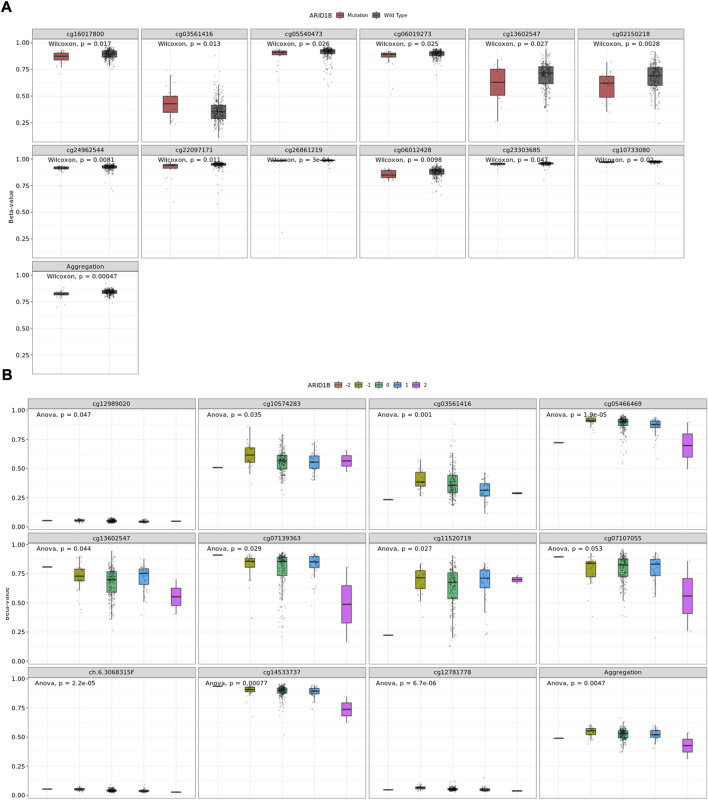
A number of CpGs are associate with ARID1B alterations **(A)** CpGs associated with somatic mutation, **(B)** CpGs associate with CNA.

The association was evaluated between somatic mutation, CNV, and DNA methylation. Results showed a number of CpG sites associated with somatic mutation and copy number variations (Figure 6A,B).

### T lymphocyte infiltrates are associated with ARID1B expression

**FIGURE 7:**
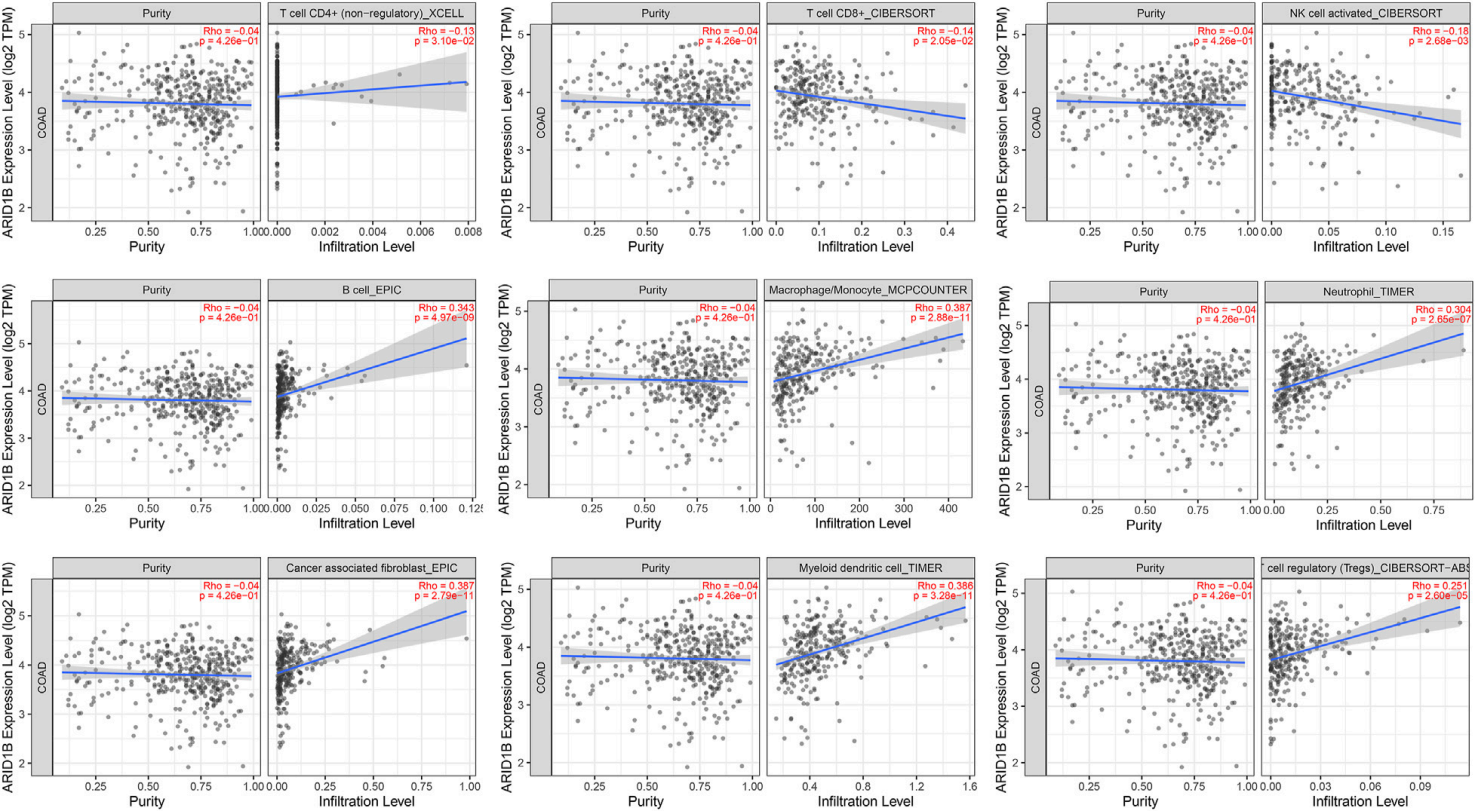
The correlation between ARID1B and immune cell infiltration. The significant negative-positive correlation between ARID1B and immune cell subtypes.

Surprisingly, as presented in Figure 7, there was a negative correlation of immune cells through TIMER analysis, including T cell, CD8^+^, T cell, CD4^+^, and NK cells and a positive correlation with B cells, macrophages, and neutrophils as well as tumor-associated fibroblast, dendritic cells, and cell regulatory cell in COAD. These data indicate that T cells CD8^+^ tend to be depleted in the COAD microenvironment and that ARID1B may play a role in the COAD microenvironment. To verify these results, we performed a Pearson correlation between markers and ARID1B expression. Strong evidence of correlation analysis was found between a variety of the biomarkers and ARID1B expression in the COAD microenvironment (Table 3). These findings emphasized that ARID1B may regulate immune cell infiltration in COAD. Dendritic cells (DCS) are key participants in the antigen-specific immune response. Two different DC subsets (XCR1, CADM1, cDC1, and CD1A) and (CD172A+ cDC2) have been identified, which interact with CD8^+^ and CD4^+^ T cells, respectively. The current study detected evidence for some DCs including XCR1, CADM1, and CD1A in COAD.

**TABLE 3:** Correlation between ARID1B and immune cell biomarkers in COAD.

Infiltrates type	Biomarker rho *P* value adj.p
T cell general	CD2 0.113171833 0.022569163 0.052159843
	CD3E 0.168758263 0.000639177 0.002480389
T cell CD8^+^	CD8A 0.079836038 0.108217539 0.191405171
B cell	CD19 0.185617928 0.000169031 0.000796579
Treg cell	CD79A 0.233264327 2.02E-06 1.40E-05
	FOXP3 0.333580951 5.22E-12 3.29E-11
	STAT5B 0.641711169 1.72E-48 8.58E-47
	TGFB1 0.226418058 4.06E-06 1.37E-05
	CCR8 0.390194392 3.25E-16 2.98E-15
Natural killer cell	KIR3DL2 0.108363891 0.029023119 0.071883887
Neutrophils	CCR7 0.299913922 6.96E-10 1.05E-08
	ITGAM 0.292339406 1.93E-09 2.41E-08
Tumor asoociated macrohage (TAM)	CCL2 0.164064315 0.000906246 0.004096026
	CD68 0.25002831 3.33E-07 2.93E-06
	IL10 0.087827792 0.07712079 0.159422822
M1 macrophage	IRF5 0.175941418 0.000368018 0.001840091
M2 macrophage	CD163 0.299282134 7.59E-10 1.10E-08
Monocyte	MS4A1 0.22754202 3.63E-06 2.62E-05
	VSIG4 0.134237336 0.006753968 0.022053772
	VEGFA 0.349636033 4.06E-13 6.50E-12
	CD86 0.197668751 6.06E-05 0.000362082
	CSF1R 0.35744943 1.11E-13 3.70E-12
Dendiritic cell	HLA-DPA1 0.144785374 0.003458199 0.010703949
	HLA-DPB1 0.130777417 0.008332108 0.022218954
	TGAX 0.313424074 1.05E-10 1.89E-09
	NRP1 0.438944503 1.50E-20 7.09E-19
	CD1C 0.217792469 9.51E-06 5.49E-05
	ITGAX 0.313424074 1.05E-10 3.13E-09
T cell exhaustion	GZMB 0.033038754 0.506791971 0.599753812
	HAVCR2 0.160201284 0.001199674 0.002864894
	CTLA4 0.19495873 7.68E-05 0.000220185
	PDCD1 0.130017099 0.00872011 0.017353452
	CD27 0.183807214 0.000196114 0.00130743
Th 1	STAT1 0.279103955 1.06E-08 4.72E-08
	STAT4 0.167092289 0.000724253 0.001788279
	TNF 0.085459081 0.085470431 0.134599104
	IFNG 0.010326018 0.835675989 0.886658875
	TBX21 0.228770674 3.20E-06 1.10E-05
Th 2	GATA3 0.295890265 1.20E-09 5.96E-09
	STAT5A 0.357832565 1.04E-13 7.79E-13
	STAT6 0.42689993 2.06E-19 2.46E-18
Th 17	STAT3 0.520078905 1.63E-29 3.63E-28
Tfh	BCL6 0.465387725 3.26E-23 4.74E-22
Fibroblast	COL1A1 0.366780269 2.25E-14 6.01E-13
	FABP5 -0.383429108 1.14E-15 3.43E-14
	NNMT 0.133761138 0.006953953 0.017754774
	PDGFB 0.424214929 3.64E-19 2.91E-17
M2 motif	BACH1 0.516162622 5.04E-29 3.25E-28
	MAF 0.423837711 3.94E-19 1.41E-18
	MAFG 0.521972992 9.41E-30 6.72E-29
	NFE2L2 0.517550043 3.39E-29 2.34E-28
	STAT2 0.461586024 8.14E-23 3.46E-22
Myloid cell	AREG 0.01689488 0.734310642 0.803393173
	CCL4 0.007207503 0.884883559 0.909905973
	CXCL2 -0.15809741 0.001394077 0.003620979
	CXCR4 0.244853698 5.89E-07 2.62E-06
	EGR1 0.304975 3.47E-10 2.24E-09
	IL1B-0.028009173 0.573612239 0.670891508
	NFKB1 0.51420058 8.83E-29 2.35E-27
	NFKB2 0.334349845 4.64E-12 3.79E-11
	NFKBIA 0.16438705 0.000885009 0.002391917
	NLRP3 0.344254286 9.72E-13 8.45E-12

### ARID1B is associated with M2 macrophages, chemokines

Since the positive correlation was found between ARID1B and TAMs biomarkers, particularly M2-macrophages, and verified these findings, we immediately studied the correlation between ARID1B and the classical chemokines of both M1-macrophages (IL-12, IL-23, TNF, IFNG) and M2 macrophages (IL-10, TGF-b, IL-4, IL-13). The analysis whose reported positive results also reported a significant positive correlation with M2 chemokines (TGF-B1, TGF-B3). Whereas none of the M1 macrophage chemokines were associated with ARID1B expression in the COAD microenvironment (Table 3). Therefore, these results support that ARID1B is closely related to M2-macrophages in the microenvironment of COAD.

### Checkpoint inhibitors are significantly correlated with ARID1B in the colon adenocarcinoma tumor microenvironment

A previous study reported that tumors may achieve immune escape by inhibiting the activity of CTLs. This is achieved by immunoinhibitory molecules that bind T cell surface ligands and inactivate its function. Therefore, we evaluated the immunomodulators level related to ARID1B expression in COAD and found a positive correlation of different immunosuppressors, including HAVCR2 (TIM3), LAG3, PDCD1 (PD-1), TIGIT, CD27, CTLA-4, and TNFRSF9 (markers of T cell failure) (Table 3). These results indicate that T cells may tend to be inactivated in the COAD microenvironment.

### ARID1B is related to the signal pathway that regulates immune cells

**FIGURE 8:**
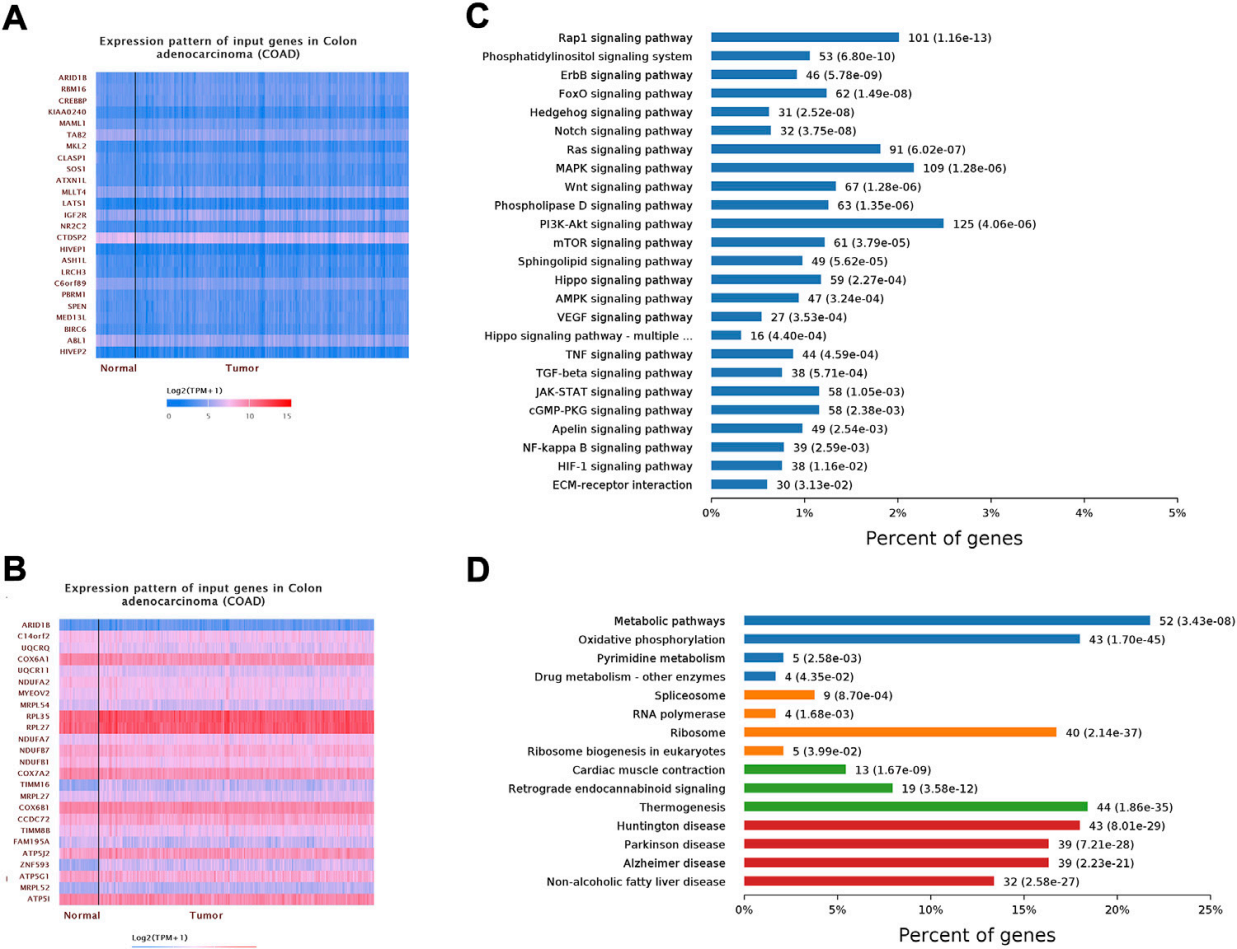
Shows gene set enrichment analysis of cooccurred genes. **(A)** Positively correlated genes **(B)** Negatively regulated genes **(C)** KEGG pathway **(D)** GO analysis.

To better understand the transcriptomic phenotype associated with infiltrated COAD tumors, ARID1B gene expression data were obtained *via* UALCAN online tool and applied gene set enrichment analysis of positive and negative correlated genes. Interestingly, RNA-seq data validated that ARID1B upregulated several immune pathways in colon cancer tissue. The enrichment results show that in ARID1B positively correlated genes, the main enrichment pathways are: (Wang et al., 2020) NF-κB signaling pathway; (Siegel et al., 2022) TNF signaling pathway; (Zhang et al., 2020a) TGF-beta signaling pathway; (Cheng et al., 2021) VEGF signaling pathway; (Han and Yoon, 2012) Wnt signaling pathway; (Zebley et al., 2021) Rap-1 signaling pathway; (Li et al., 2014) PI3K-Akt signaling pathway; (Zhou et al., 2019) mTOR signaling pathway; (Watts et al., 2008) JAK-STAT signaling pathway innate immune response; (Salta et al., 2018) ECM receptor interaction. The main enrichment pathways in ARID1B negatively associated genes were: (Wang et al., 2020) metabolic pathway; (Siegel et al., 2022) RNA polymerase, Huntington disease, Parkinson disease, Alzheimer disease; (Zhang et al., 2020a) Thermogenesis; (Cheng et al., 2021) non-alcoholic fatty liver disease; (Han and Yoon, 2012) spliceosome; (Zebley et al., 2021) oxidative phosphorylation; (Li et al., 2014) retrograde endocannabinoid signaling; (Zhou et al., 2019) oxidative phosphorylation (Figures 8A–D).

## Discussion

Among the different types of molecule abnormalities, mRNA changes and DNA methylation, which are reported widely in almost all cancers. The main purpose of this study was to analyse the promotor methylation of ARID1B through preliminary difference analysis, correlation analysis, and prognosis analysis. Despite very little was found in the literature on the analysis of ARID1B expression and its DNA methylation in cancer, none of these studies was related to colon cancer (Aso et al., 2015; Cui et al., 2019; Tessier-Cloutier et al., 2020). Previous studies observed an increased expression of ARID1B in triple-negative breast cancer and breast invasive ductal carcinoma (Shao et al., 2015; Cui et al., 2019). In the current study, we evaluated DNA methylation and gene expression profiles of ARID1B in colon cancer samples from TCGA. The expression of ARID1B in TCGA COAD and normal tissues were first analysed using the TNMplot platform. To confirm the observed results experimentally, RNA from the colon cell line was extracted and detected by qPCR analysis. This method was repeated three times to ensure reliable results. Consistent with the significant downregulation of ARID1B expression in colon cell lines and TCGA tissues, ARID1B was hypermethylated in COAD tissues. Comparison of these findings with those of previous studies confirmed silenced expression of genes, especially ARID1B by aberrant methylation of DNA in cancer (Chuang and Jones, 2007; Khursheed et al., 2013; Liu et al., 2020; Tan et al., 2020). AKR1C2 mRNA, a gene previously identified as a Nrf2-target, was not found in HCT116 cells (Ebert et al., 2011). Importantly, survival analysis showed that the overall survival rate of patients in the low mRNA expression group was significantly shorter than that in the low-level group. ARID1B methylation level was related to clinicopathological characteristics and showed a significant negative correlation with its mRNA level in COAD. Besides, the prognostic analysis of a number of methylated sites was significantly associated with the patients’ outcome in COAD patients. These consistent results of aberrantly silenced ARID1B provide strong evidence that low ARID1B expression is an important indicator of the poor prognosis of COAD and the epigenetic changes might be a potentially increased risk of colon cancer-related death. Knowing that DNMTs enzymes are responsible for establishing, maintaining, and mediating DNA methylation, we performed correlation analysis, and ARID1B was positively correlated with DNMTS in COAD (Chuang and Jones, 2007). The third question in this research aimed to determine the pathways of ARID1B expression on the basis of TCGA database. GSEA revealed that ARID1B is involved in multiple cancer-related pathways, among others PI3K-AKT and mTOR pathways, suggesting that ARID1B regulates these pathways and functions as a tumour suppressor in COAD.

Tumour infiltrates immune cells are an important immune cell type in the tumor microenvironment and have been reported to affect patients’ overall survival (Kryczek et al., 2016; Yang et al., 2018). For this and to further determine the association between ARID1B and COAD, we used TIMER database to analyse the correlation between the expression of ARIDIB and immune cell infiltration. Immune cells and fibroblast infiltration demonstrated a link with ARID1B in the COAD microenvironment. Consistent with the obtained results, ARID1B showed a positive correlation with immune cell markers. It was reported that CD8^+^ T cells could be inhibited by immunoinhibitory molecules *via* binding to the receptor PD-1 on the surface of CD8^+^ T cells (Yang et al., 2018). TIMER analysis revealed a positive correlation between ARID1B and T-exhausted cell immunoinhibitory molecules. The positive correlation of ARID1B and macrophages and depleted CD8^+^ markers indicates the contribution of macrophages to the formation of the immunosuppressive microenvironment in COAD. Recently, The M2-polarization of TAMs was recognized as an immunosuppressive phenotype (Mu et al., 2018). Moreover, scientists found that monocytes recruited to tumour regions are reprogrammed to become tumour-associated macrophages. And the activity of motifs MAF, STAT1, STAT2 leads to M2 polarization, which provides potential targets for inhibiting or reversing the information of the immunosuppressive microenvironment (Zheng et al., 2021). In addition to TILs, ARID1B was positively correlated with TIM, including LAMP3, cDCs, LYVE1, resident tissue macrophages (RTMs), and FOLR2, TAM. In accordance with this result, recent work reported multiple tumour types of TIM cell subpopulations across the pan-cancer analysis (Cheng et al., 2021).

Finally, we explored the signal pathways that might regulate the immune cell infiltration and polarization of M2 macrophages in COAD. TGF-β signalling pathway is a cytokine signalling pathway involved in the development and progression of COAD. Hu et al. found that the transcription factors STAT3, HIVEP, NFAT, and other regulated genes are upregulated in depleted CD8^+^ T cells. This result provides clues for identifying new candidate transcription factors for T cell dysfunction. Furthermore, STAT3 not only participates in the polarization of macrophages to M2 but also participates in the depletion of T cells, suggesting that STAT3 inhibition can be used as a new therapeutic strategy for the treatment of cancer (Hu et al., 2020). A pathway enrichment analysis of ARID1B-correlated genes also indicated the involvement of the STAT-JAK signalling pathway, which suggest that the TGF-β1/STAT-JAK pathways are associated with aggressive pathological features and poor clinical outcomes in COAD. While this report describes the function of ARID1B and its methylation in COAD, it also provides preliminary evidence about the role of ARID1B in the microenvironment of COAD immune cell infiltration, and M2 polarization and suggests that ARID1B may influence COAD immunotherapy. Our study has some limitation. The involvement of ARID1B in COAD was identified *via* bioinformatics. Even though the association between ARID1B and DNMTS expressions in COAD was not statistically significant, it remained unknown whether ARID1B was regulated by DNTMs enzymes. However, future experimental studies on the current topic are therefore recommended. In conclusion, the article comprehensively analysed ARID1B abnormalities (mRNA changes, DNA aberrant methylation) and its association with the immune cell infiltration of COAD. The analysis revealed that ARID1B hypermethylation could serve as an early diagnostic biomarker for COAD treatment, and the difference in immune cell infiltration was found to be related to the ARID1B expression of the COAD tumour. In conclusion, the article comprehensively analysed ARID1B abnormalities (mRNA changes, DNA aberrant methylation) and its association with the immune cell infiltration of COAD. The analysis revealed that ARID1B hypermethylation could serve as an early diagnostic biomarker for COAD treatment, and the difference in immune cell infiltration was found to be related to the ARID1B expression of the COAD tumour.

## Data Availability

The original contributions presented in the study are included in the article/Supplementary Material, further inquiries can be directed to the corresponding authors
